# Comparison of Four Spatial Interpolation Methods for Estimating Soil Moisture in a Complex Terrain Catchment

**DOI:** 10.1371/journal.pone.0054660

**Published:** 2013-01-23

**Authors:** Xueling Yao, Bojie Fu, Yihe Lü, Feixiang Sun, Shuai Wang, Min Liu

**Affiliations:** State Key Laboratory of Urban and Regional Ecology, Research Center for Eco-Environmental Sciences, Chinese Academy of Sciences, Beijing, P. R. China; NASA Jet Propulsion Laboratory, United States of America

## Abstract

Many spatial interpolation methods perform well for gentle terrains when producing spatially continuous surfaces based on ground point data. However, few interpolation methods perform satisfactorily for complex terrains. Our objective in the present study was to analyze the suitability of several popular interpolation methods for complex terrains and propose an optimal method. A data set of 153 soil water profiles (1 m) from the semiarid hilly gully Loess Plateau of China was used, generated under a wide range of land use types, vegetation types and topographic positions. Four spatial interpolation methods, including ordinary kriging, inverse distance weighting, linear regression and regression kriging were used for modeling, randomly partitioning the data set into 2/3 for model fit and 1/3 for independent testing. The performance of each method was assessed quantitatively in terms of mean-absolute-percentage-error, root-mean-square-error, and goodness-of-prediction statistic. The results showed that the prediction accuracy differed significantly between each method in complex terrain. The ordinary kriging and inverse distance weighted methods performed poorly due to the poor spatial autocorrelation of soil moisture at small catchment scale with complex terrain, where the environmental impact factors were discontinuous in space. The linear regression model was much more suitable to the complex terrain than the former two distance-based methods, but the predicted soil moisture changed too sharply near the boundary of the land use types and junction of the sunny (southern) and shady (northern) slopes, which was inconsistent with reality because soil moisture should change gradually in short distance due to its mobility in soil. The most optimal interpolation method in this study for the complex terrain was the hybrid regression kriging, which produced a detailed, reasonable prediction map with better accuracy and prediction effectiveness.

## Introduction

Soil moisture (SM) is of fundamental importance in meteorology, agriculture, and hydrology, among other scientific disciplines [1], [2], [3], [4]. In hydrology, SM partitions rainfall into runoff and infiltration, thus impacting the surface and groundwater recharge, flood forecasting, and flow routing modeling [5], [6]. Scientists usually need accurate, spatially continuous data across a region in order to make justified interpretations, but such data are usually not readily available and are often difficult and expensive to acquire. Remote sensing techniques have great potential for measuring spatially continuous SM data, but typically involve observing the average SM close to the ground surface and over large geographical areas with low resolution [7]. Many approaches combining remotely sensed data and auxiliary model to estimate deeper soil moisture have been developed, such as infiltration models [8] and knowledge based techniques that use prior information of hydrology [9] and so on. However, these approaches cannot meet the requirement of small catchment scale researches, which need finer spatial resolution of deep soil moisture data to study the exchange of water between different layers within the soil column or between the land surface and the atmosphere [10]. In situ measurements of soil moisture are still an important portion in recent researches [5], [11], [12]. The popular in situ techniques of measuring soil moisture content include gravimetric method, neutron probes, electromagnetic techniques, cosmic-ray neutrons and so on [13]. However, theses in situ techniques typically involve measuring SM in points. Spatially continuous SM data in deep soil with finer resolution are needed in many cases. Thus, estimating the values at unsampled sites using data from point observations is necessary, and spatial interpolation methods provide an essential tool to meet this need.

In previous research, the geostatistical method (ordinary kriging (OK), cokriging) [14], [15], [16], geometric method (inverse distance weighting (IDW), local polynomial), and statistical methods such as the linear regression model (LR) [17], [18], [19] have been the most commonly used interpolation technologies [1], [20]. In addition, hybrid interpolation techniques, which combine two conceptually different approaches, have received increasing attention in recent years [21], [22]. One of these techniques is known as regression kriging (RK) [23], [24], and first uses regression on auxiliary information and then uses simple kriging (SK) with a known mean (0) to interpolate the residuals from the regression model [25]. Zhu [23] compared the performance of OK and RK for soil properties in different landscapes and indicated that when a strong relationship existed between the target soil properties and auxiliary variables and the terrain was relatively complex, RK was more accurate for interpolating soil properties. Li and Heap [26] investigated the performance and impact factors of popular interpolation methods in environmental sciences by accessing 53 comparative studies. The results indicated that the OK and IDW methods were the most frequently used. The performance of a spatial interpolation method depends not only on the features of the method itself, but also on factors such as data variation and sampling design. Most of the methods performed at an acceptable level for predicting soil properties in a gentle terrain [27], [28], but few performed well in a complex terrain.

Our research was conducted in the semi-arid Loess Plateau, which has a complex hilly gully terrain. The SM in the deep soil was paid much attention because it significantly affects the growth of the planted vegetation as well as the success of the Grain for Green Project (a state campaign in China to restore an ecological balance to the country’s western parts, by turning the low-yielding farmland back into forests and pasture) in this region [29], [30]. Due to the limitation of remote sensing technology for directly obtaining deep SM with fine resolution [7], [31], many studies in the Loess Plateau have been based on ground and point measurements [32], [33], [34], [35]. However, considering the intensive labor consumption and destruction to the ground when conducting sampling, the sample density is usually insufficient at the catchment or region scale. Based on these practical challenges, we aimed to determine an optimal interpolation method that fits to the hilly gully terrain in the semi-arid Loess Plateau.

Two of the most popular interpolation methods, OK and IDW, were chosen in our research [26], [33], [36], [37], as investigating their suitability in complex terrain is practically valuable to further research. Secondly, considering the SM in the Loess Plateau was strongly impacted by geographic factors such as land use type [38], [39], soil properties [35], gradients, slope aspects [40], and so on, the LR model was chosen, in which the strong correlation between SM and geographic factors would be helpful to create an optimal regression function [26]. A hybrid RK model was also chosen for its theoretical suitability in our research and good performance in previous research [23], [41], [42], [43]. The performance of each method was assessed in terms of mean-absolute-percentage-error (MAPE), root-mean-square-error (RMSE), and goodness-of-prediction statistic (G). The theoretical and practical advantages and disadvantages of each method for a complex terrain are discussed in detail at the end of this research.

## Study Area

**Figure 1:**
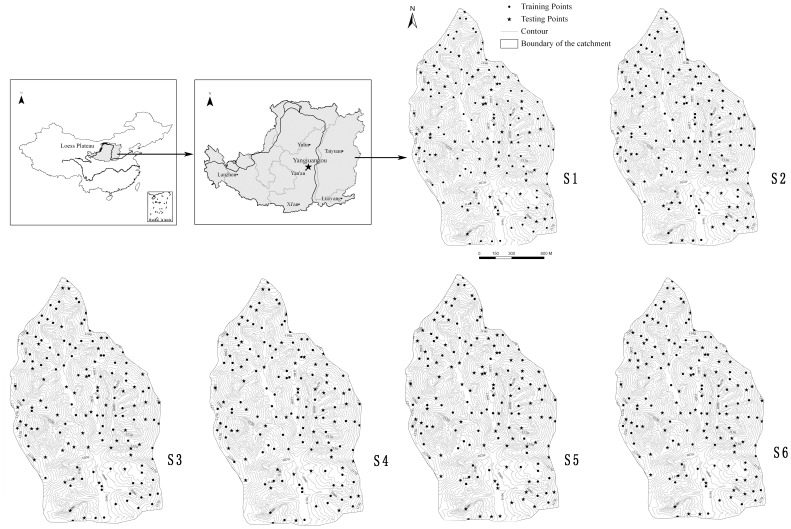
The location of the study catchment and the distribution of the samples.

The study was carried out in the Yangjuangou catchment (36°42′N, 109°31′E), which is located in the center of the Loess Plateau near Yan An City in Shaanxi Province, China (Fig. 1). The catchment has a total area of 2.02 km^2^ and the elevation ranges from 1,050 to 1,298 m. It is a typical gully and hilly area with a gully density of 2.74 km km^− 2^ and the slope gradients range from 10° to 30°. The area has a semi-arid continental climate with an average annual rainfall of 535 mm. Rainfall events occur mainly between June and September with large inter-annual variation. The soil in the study area was derived from loess with a maximum depth of approximately 200 m. The soil texture was rather homogeneous, containing mainly loessial sandy loam soil with the silt particles being 65–75% and bulk density being 1.2–1.4 g/cm^3^ according to laboratory measurements. As result of the Grain for Green project that was launched in 1998, most of the cultivated lands on steep slopes were abandoned for natural or planted vegetation. Grasslands and forestlands now dominate the hillslopes, and shrubs are thriving at the bottom of the north-facing slopes. The main forest species in the Yangjuangou catchment is *acacia* (*Robinia pseudoacacia*), which was planted in the 1980s and after 1999. The dominant grass species are *Artemisia sacrorum*, *Stipa bungeana*, and *Artemisia scoparia*. The main shrub species are *Prunus armeniaca* and *Hippophae rhamnoide*. A mosaic of patchy land cover is the typical landscape pattern in the Yangjuangou catchment as a result of human disturbances as well as climatic and topographical conditions [44].

## Experimental Layout and Methods

### Experimental Layout

The SM measurement was conducted in June 2010, with a total of 153 points being measured. The slope distances between neighboring points ranged from 50 m to 100 m and probably met the uniform distribution, which was required by Geostatistical interpolation methods. Besides, all the land use types and typical topography types in the catchment were involved and each land use/topography type contained at least ten sampling points to ensure the validity when conduct statistical analysis. The field sample collection lasted three days, and there was no rain during these days. The distribution of the points was shown in Figure 1. The soil samples in 10 cm, 20 cm, 40 cm, 60 cm, 80 cm and 100 cm depth were extracted using a soil auger at each point. Once extracted from the ground, the samples were placed in aluminum cans with tight-fitting lids, and the gravimetric water content was determined from the weight loss after oven drying at 105°C to a constant mass. The soil bulk density was measured synchronously in each plot using a ring cut. The volumetric water content was calculated by multiplying the gravimetric water content and the soil bulk density and dropping the units. The soil moisture in 10–20 cm layers was averaged to present the upper soil layer for each point, while the 40–100 cm to present the deeper soil layer.

In the field, a GPS receiver with 5 m precision was used to obtain the altitude, longitude, and latitude, which were later imported into a geographic information system (ArcGIS 9.3) as Albers coordinates. The slope degree and aspect were measured with a geological compass. The land use types, primary soil types, and vegetation species and coverage (%) were estimated by observation.

To produce a spatially continuous surface and evaluate the performance of each interpolation method, the 153 sampling points were randomly divided into two sets by the “Create Subset” component of the Geostatistical Analyst extension in ArcGIS: the training data set and the test data set with 2∶1 ratio. The training data set was used to create the model and the test data sets were used to assess the performance of the model. In the present study, the training data set included 102 points and the test data set included 51 points. Considering that the distribution of the training points may affect model performance, the subsetting was repeated six times (S1 to S6). For each subset, the four interpolation methods were used to produce the spatially continuous surface, and the performance was assessed accordingly.

### Methods

For the IDW and OK interpolation methods, the value of variable Z at the unsampled location x_0_, Z*(x_0_) is estimated based on the data from the surrounding locations, Z (x_i_), as
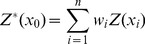
(1)where *w_i_* is the weight assigned to each Z (x_i_) value and *n* is the number of the closest neighboring sampled data points used for estimation. The weights for the IDW are usually proportional to the inverse of the squared distance between the prediction point and the observation points, and they sum to 1. That is,
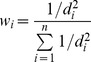
(2)where di is the distance between the estimated point and the observed point.

The number of the closest neighboring samples is an important factor affecting the precision of IDW. Considering the sample spatial density and the hilly gully terrain of our research area, the number of the closest neighboring samples should be small because the samples taken on the other side of the hill should have little correlation with the predicted point in reality. Thus, the number of the closest neighboring samples we applied varied from 3 to 6. Cross-validation was used to compare the results obtained with a different number of the closest neighboring samples. The numbers of the closest neighboring samples producing the best agreement between the observed data and the estimates were chosen as the optimal IDW weighting parameters [20].

Kriging calculates the values of *w_i_* by estimating the spatial structure of the variable’s distribution represented by a sample variogram as
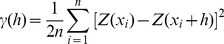
(3)where *x_i_* and *x_i_*+*h* are sampling locations separated by a distance *h*, and *Z*(*x_i_*) and *Z*(*x_i_*+*h*) are the observed values of variable *Z* at the corresponding locations. The sample variogram is fitted with a variogram model and the adequacy of the chosen model is tested using cross-validation. In this study, we considered the spherical, Gaussian, and exponential models for the sample variogram fitting. The cross-validation was conducted with varying model parameter values and the numbers of the closest neighboring samples ranging from 3–10 until the highest estimation accuracy was reached.

Linear regression is a common forecasting tool for many research areas. It is a statistical tool for modeling the relationship between a dependent variable and one or more independent variables. In linear regression models, the dependent variable is a linear function of one or more independent variables, as shown in the equation below.
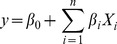
(4)


The parameters of the linear regression model are typically estimated using the least squares method, which results in a line that minimizes the sum of squared vertical distances from the observed data points to the line [19].

A large body of research indicates that the SM in the Loess Plateau is strongly affected by land use types, soil properties, and terrain [11], [40], [45], [46], [47]. Considering the soil properties were fairly homogeneous in our research area (mainly loessial sandy loam soil with the silt particles being 65–75% and bulk density being 1.2–1.4 g/cm^3^ basing on laboratory detection), the independent variables were selected as land use types, slope, and annual average solar radiation, which were preliminary detected basing on correlation analysis and finally determined basing on the significance of regression coefficient (P<0.05) in the regression equation. The slope and annual average solar radiation were continuous variables, which were produced by digital elevation model (DEM, 3 m×3 m). The land use types were categorical variables and were converted into dummy variables before they were introduced into the regression analysis. When all of the parameters of the linear function were produced and satisfied the significance test (*P*<0.05) in SPSS 13.0 software, they were adopted to produce the prediction map in ArcGIS 9.3.

RK is a spatial interpolation technique that combines the regression of the dependent variable on auxiliary variables with the kriging of the regression residuals. The target variable SM, was fitted with each auxiliary data set using the linear regression. By detrending the regression predictions, the residuals were geostatistically analyzed and interpolated using SK, and finally the regression predictions and interpolated residuals were summed. Eventually, the SM predictions were back-transformed to normal SM values. In RK, the auxiliary data sets in the regression were the same as in the LR [25].

**Figure 2:**
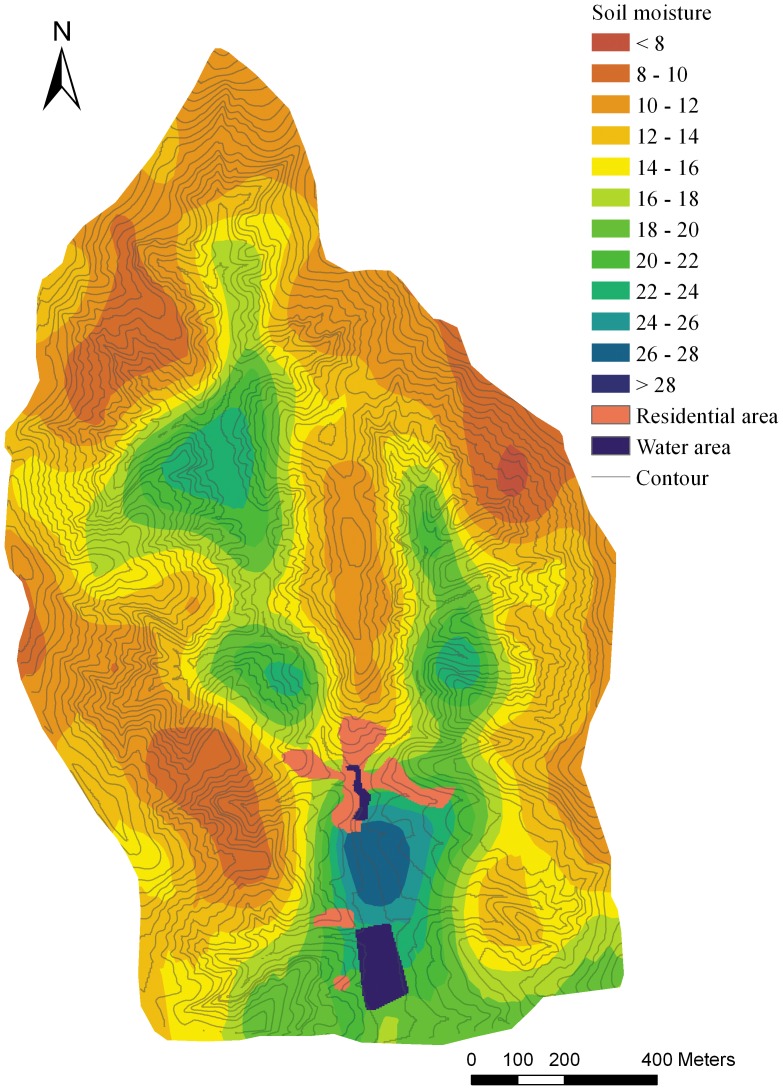
The soil moisture prediction map basing on ordinary kriging method.

**Figure 3:**
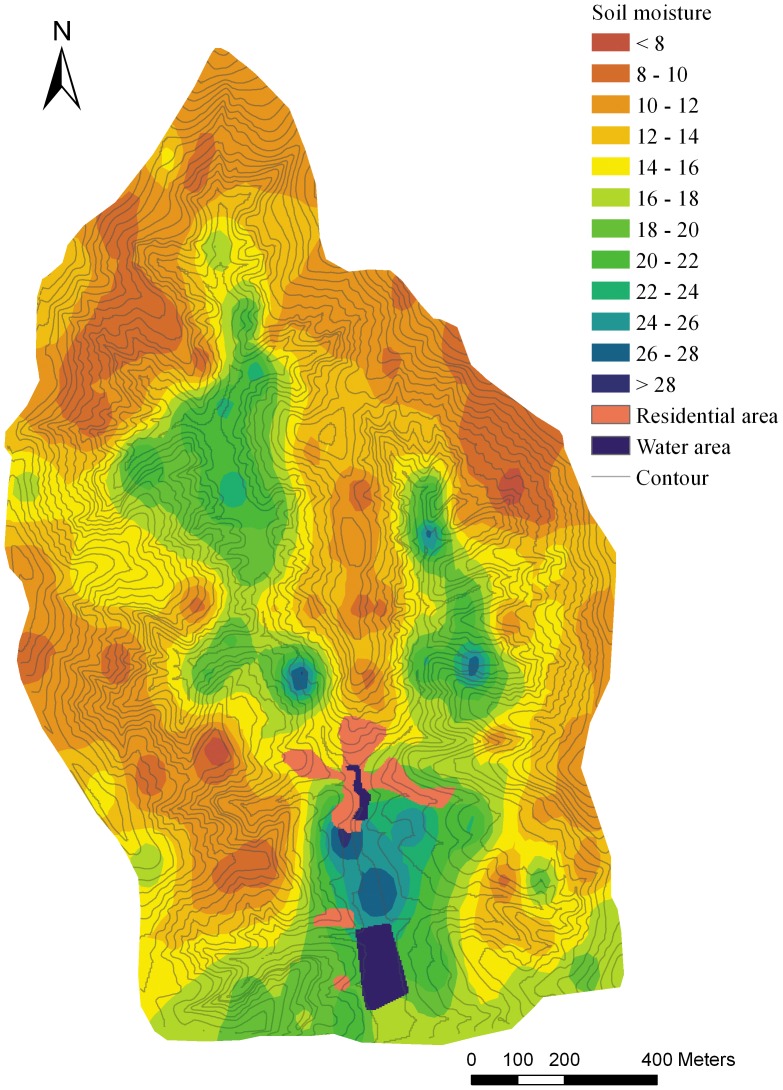
The soil moisture prediction map basing on inverse distance weighting method.

**Figure 4:**
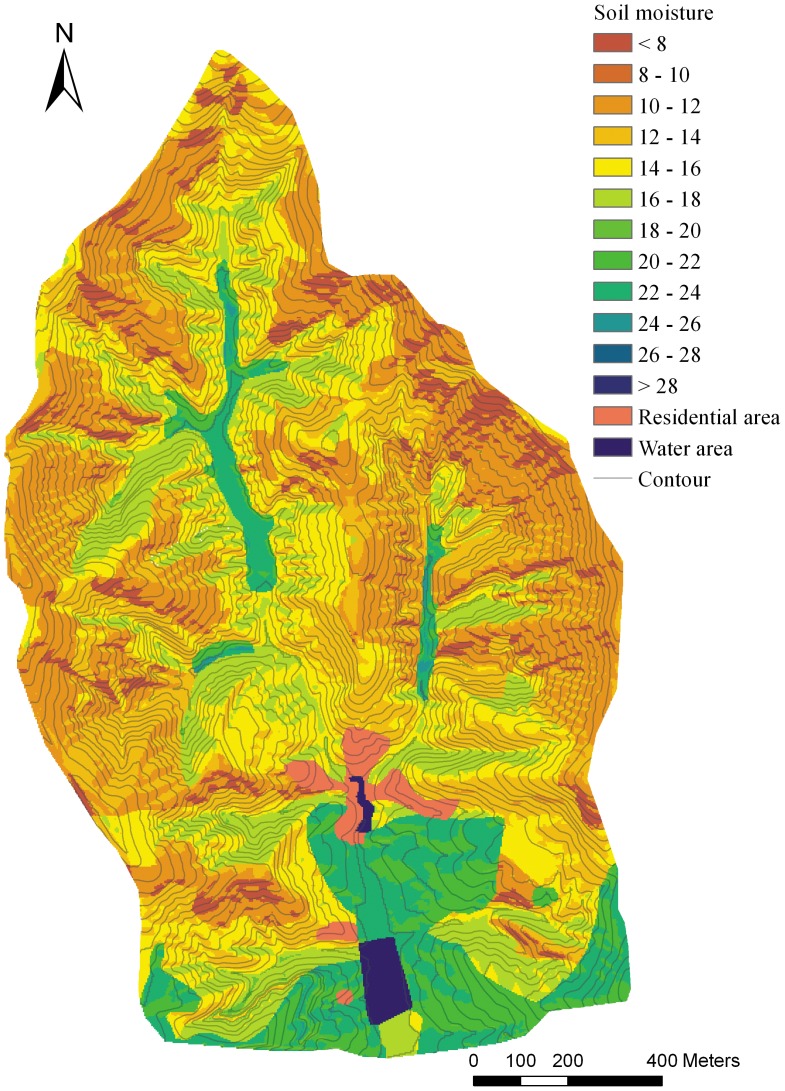
The soil moisture prediction map basing on linear regression method.

**Figure 5:**
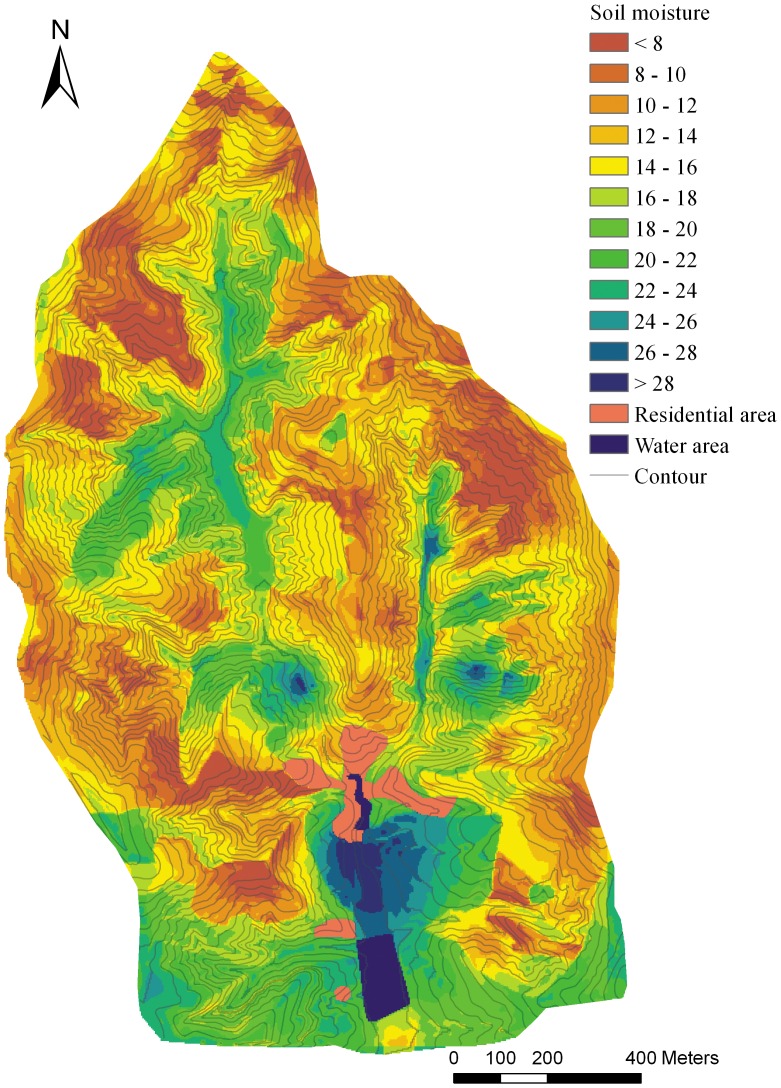
The soil moisture prediction map basing on regression kriging method.

The IDW and OK method were conducted using ArcGIS 9.3. With the LR and RK methods, the linear regression function was established by SPSS 13.0 and the prediction of the continuous spatial surface was conducted by ArcGIS. Eventually, all of the predicted maps were laid out using ArcGIS with comparable design (Fig. 2, 3, 4, 5).

### Assessment of Method Performance

The performance of the methods was assessed by identifying the error in the predictions. For each method, the prediction values and corresponding observed values in the test data set were compared, and the following evaluation indicators were calculated.

The accuracy was measured by MAPE, which is an accuracy measure based on percentage (or relative) errors and RMSE, which measures the average magnitude of the error [26]. The errors are squared before they are averaged, so the RMSE gives a relatively high weight to large errors. This means that RMSE is most useful when large errors are particularly undesirable. Small MAPE and RMSE values indicate a model with few errors and more accurate predictions.

The MAPE is calculated as follows:
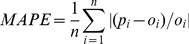
(5)


Where *n* is the number of validation points, *p_i_* is the predicted value at point *i*, *o_i_* is the observed value at point *i.*

The RMSE is calculated as follows:
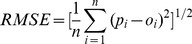
(6)


Where *n* is the number of validation points, *p_i_* is the predicted value at point *i*, *o_i_* is the observed value at point *i.*

The effectiveness of the models was evaluated using a goodness-of-prediction statistic (G). The G-value measures how effective a prediction might be relative to that which could have been derived using the sample mean:
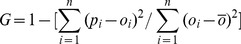
(7)


Where *n* is the number of validation points, *p_i_* is the predicted value at point *i*, *o_i_* is the observed value at point *i*, and *ō* is the sample arithmetic mean. A G-value equal to 1 indicates perfect prediction, a positive value indicates a more reliable model than if the sample mean had been used, a negative value indicates a less reliable model than if the sample mean had been used, and a value of zero indicates that the sample mean should be used [20].

## Results

### Basic Statistics of Soil Moisture

The SM of the overall samples ranged from 5.49% to 35.94%, with an average value of 14.08%. The SM spatial variability was significant, with a standard deviation (Std.dev) of 5.85 and coefficient of variation (CV) of 0.42 (Table 1). The skewness values of the data sets were positive and the asymmetry was obvious, with the value of 1.2. After the log-transformation, the corresponding skewness values were much smaller (0.39), which means that the data distribution was closer to normal. The log-transformed data sets were used in the Kriging interpolation method, which strictly demands normality of the data set [48]. The statistics values of each subset (S1 to S6) were also listed in Table 1.

**Table 1:** The basic statistical properties of soil moisture of each data set.

	Count	Min	Max	Average	Std.dev	Skewness	Kurtosis	CV
					O/L	O/L	O/L	O/L
All	153	5.49	35.94	14.08	5.85/0.38	1.16/0.39	3.95/2.44	0.42/0.15
S1	102	5.49	28.59	13.96	5.57/0.38	0.82/0.25	2.67/2.12	0.40/0.15
S2	102	6.44	35.94	14.20	6.19/0.40	1.22/0.47	4.03/2.41	0.44/0.16
S3	102	6.60	35.94	14.03	5.96/0.39	1.21/0.53	4.02/2.36	0.42/0.15
S4	102	5.49	35.94	14.42	6.02/0.39	1.19/0.33	4.11/2.62	0.42/0.15
S5	102	5.49	29.16	13.70	5.47/0.37	1.16/0.43	3.70/2.55	0.40/0.15
S6	102	6.44	35.94	14.07	5.82/0.38	1.20/0.42	4.23/2.44	0.41/0.15

O. Statistical value from Ordinary dataset.

L. Statistical value from Log-transformed dataset.

### Correlation between the Sample Pattern and the Performance of the Interpolation Methods

The Std.dev, skewness, kurtosis, and CV statistics were used to represent the pattern properties of each subset. The Pearson Correlation between the pattern properties and the G-values were used to analyze the effect of the sample pattern on the performance of each method. Table 2 shows that all of the correlations were poor and none of the correlation coefficients were significant in this study.

**Table 2:** The correlation between the G-values and the sample pattern properties basing on correlation analysis.

	Std.dev		Skewness		Kurtosis		CV	
Method (soil depth, cm)	PCC	P	PCC	P	PCC	P	PCC	P
OK (10–20)	−0.75	0.08	−0.68	0.14	−0.73	0.10	−0.74	0.09
IDW (10–20)	−0.70	0.12	−0.45	0.37	−0.39	0.44	−0.85	0.03
LR (10–20)	−0.44	0.38	−0.32	0.54	−0.40	0.43	−0.44	0.38
RK (10–20)	−0.60	0.21	−0.54	0.27	−0.56	0.25	−0.68	0.14
OK (40–100)	−0.53	0.27	−0.12	0.82	−0.09	0.86	−0.67	0.15
IDW (40–100)	−0.59	0.22	−0.20	0.70	−0.14	0.79	−0.74	0.09
LR (40–100)	−0.76	0.08	−0.23	0.66	−0.28	0.59	−0.80	0.06
RK (40–100)	−0.67	0.14	−0.27	0.60	−0.30	0.57	−0.75	0.08

PCC. Pearson correlation coefficient.

P. Significance value (2-tailed).

### Performance of the Interpolation Methods

The MAPE (RMSE) were generally decreasing from OK to RK in Table 3 and Table 4, with the corresponding average values of 0.36 (4.04), 0.33 (3.74), 0.23 (2.70), 0.20 (2.72) for the 10–20 cm layer and 0.33 (5.10), 0.32 (4.84), 0.25 (4.20), 0.25 (4.11) for the 40–100 cm layer, which indicated a greater probability that errors occur in OK and IDW than in LR and RK.

**Table 3:** The performance assessment of the four interpolation methods for 10–20 cm soil layer.

	MAPE				RMSE				G-value			
	OK	IDW	LR	RK	OK	IDW	LR	RK	OK	IDW	LR	RK
S1	0.28	0.29	0.19	0.15	3.65	3.58	2.68	2.28	0.54	0.56	0.75	0.82
S2	0.36	0.37	0.26	0.24	3.77	3.86	2.64	2.99	0.17	0.13	0.59	0.56
S3	0.40	0.35	0.22	0.19	4.40	3.69	2.72	2.53	0.24	0.47	0.71	0.75
S4	0.28	0.25	0.21	0.19	3.61	3.27	2.13	2.29	0.42	0.53	0.80	0.83
S5	0.41	0.43	0.20	0.24	5.01	4.88	3.16	3.43	0.50	0.53	0.80	0.83
S6	0.39	0.29	0.27	0.22	3.77	3.15	2.85	2.79	0.20	0.44	0.54	0.56
Average	0.36	0.33	0.23	0.20	4.04	3.74	2.70	2.72	0.35	0.44	0.70	0.69

**Table 4:** The performance assessment of the four interpolation methods for 40–100 cm soil layer.

	MAPE				RMSE				G-value			
	OK	IDW	LR	RK	OK	IDW	LR	RK	OK	IDW	LR	RK
S1	0.36	0.33	0.26	0.25	5.82	5.44	4.72	4.50	0.28	0.37	0.53	0.57
S2	0.28	0.29	0.23	0.23	4.03	4.09	3.54	3.84	0.09	0.06	0.30	0.28
S3	0.36	0.34	0.23	0.23	4.99	4.93	3.80	3.50	0.26	0.28	0.57	0.64
S4	0.29	0.26	0.28	0.26	4.91	4.47	4.47	4.21	0.40	0.50	0.50	0.55
S5	0.41	0.40	0.26	0.27	5.91	5.49	4.40	4.18	0.37	0.45	0.65	0.68
S6	0.27	0.27	0.26	0.26	4.97	4.64	4.25	4.41	0.26	0.36	0.46	0.42
Average	0.33	0.32	0.25	0.25	5.10	4.84	4.20	4.11	0.28	0.34	0.50	0.51

The G-value reflects the prediction effectiveness of each method. For the S1 to S6 subsets, the G-values were ranked as OK<IDW<LR<RK, with corresponding average values being 0.35, 0.44, 0.70, 0.69 for the 10–20 cm soil layer and 0.28, 0.34, 0.50, 0.51 for the 40–100 cm soil layer. According to the multiple mean comparison (Student-Newman-Keuls) of G-value of each method, the four interpolation methods were divided into two classes. The OK and IDW methods were classed into a same group because the G-values were not significantly different, while the LR and RK were classed into another group (Table 5, Table 6). The G-values difference between the two classes was significant (*P*<0.05), which means that the effectiveness of the LR and RK methods was significantly better than the distance-based OK and IDW methods. For OK and IDW, the G-value of data set S2 for 40–100 cm soil layer was close to 0, which indicates that the effectiveness of the prediction was not better than if the sample mean was used [20]. Although the relatively better performance of IDW than OK, both of them were not optimal in complex terrain because of their larger error and lower prediction effectiveness. Comparatively, the performances of LR and RK were much better, with the average G-values about 0.7 for 10–20 cm soil layer and 0.5 for 40–100 cm soil layer. Although the G-value of LR was higher than RK in several cases, the RK performance was generally better than LR in terms of all the three assessment indicators (Table 3, Table 4).

**Table 5:** The means comparison of the G-value of the four interpolation methods for 10–20 cm soil layer.

		Sub-classification for *P* = 0.05	
Method	Sample Number	1	2
OK	6	0.35	
IDW	6	0.44	
LR	6		0.70
RK	6		0.73
P		0.25	0.75

P. Significance value.

**Table 6:** The means comparison of the G-value of the four interpolation methods for 40–100 cm soil layer.

		Sub-classification for *P* = 0.05	
Method	Sample Number	1	2
OK	6	0.28	
IDW	6	0.34	
LR	6		0.50
RK	6		0.52
P		0.45	0.06

P. Significance value.

## Discussion

Many researchers indicated that data normality [48] and CV [26] might affect the performance of spatial interpolation methods. In our research, the S1 to S6 subsets corresponded to six different sample patterns. For each interpolation method, the prediction errors between the six patterns obviously differed, indicating that the sample pattern may significantly affect the performance of the methods, which has been referred in many studies [49], [50]. However, from the correlation analysis, we did not find any significant correlation between the sample pattern properties and the G-values (Table 2). The factors inducing the performance difference between each sample pattern is still not clear in this study and need to be analyzed in further research.

**Figure 6:**
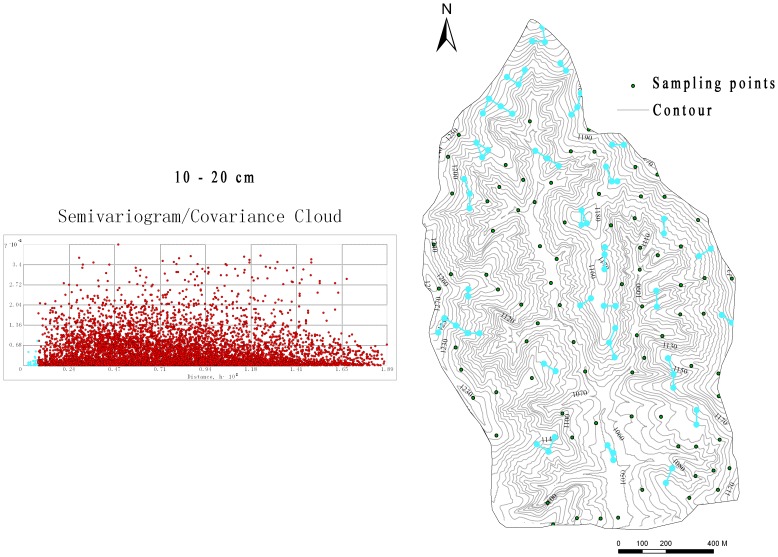
The autocorrelation range of the 10–20 cm soil moisture in complex terrains.

**Figure 7:**
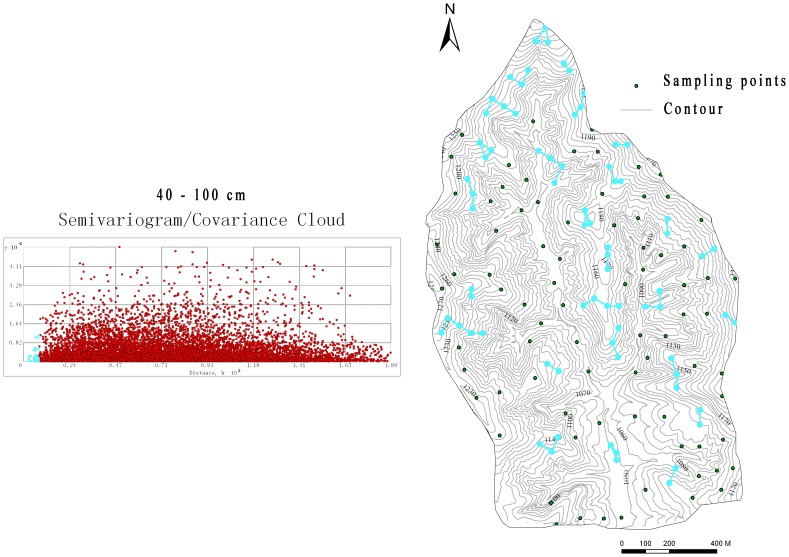
The autocorrelation range of the 40–100 cm soil moisture in complex terrains.

The spatial autocorrelation of the target variable is a basic assumption for the distance-based interpolation method. The spatial autocorrelation is present when the value of a variable at one location exerts an influence on the value of the same variable in neighboring locations [51]. Theoretically, the spatial autocorrelation of SM should exist at a small scale because of the water mobility in soil. However, SM may have little autocorrelation at small catchment scale because of the strong control of local geographical factors to SM [11]. Climate could be seen as homogeneous in small catchment scale, thus the local factors, such as land use types, vegetation, soil types and topography therefore stood out as the main factors dominating the soil moisture spatial distribution. Because of the spatial fragmentation of these factors in hilly-gully area, the soil moisture may show poor spatial autocorrelation. The semivariogram cloud confirmed the short-distance autocorrelation of the SM in this complex terrain area (Fig. 6, Fig. 7), showing that spatial autocorrelation only exists at slope scale (<100 m) and no obvious autocorrelation exists at small catchment scale. One of the basic assumptions of ordinary kriging was that the observations have obvious spatial autocorrelation and the autocorrelation is a function of the distance between the observations [50]. Obviously, the short autocorrelation range of SM in this area was a lack of statistical effectiveness, which could hardly fit this assumption. Thus, it is not difficult to understand the poor performance of OK in the present research (Fig. 3). For IDW, the performance was slightly better than OK because it simply relies on the similarity of neighboring sample points to predict the unmeasured points. However, the IDW still did not perform its best because of the limitation of the low sampling density relating to the complex terrain (Fig. 4).

Although the SM in upper soil was more sensitive to ground impact factors (such as weather) and was expected to have better spatial autocorrelation at catchment scale, the results of the semivariogram analysis (Fig. 6) showed little difference with that in the deeper soil (Fig. 7). The performance of the four methods for the upper soil moisture generally appears better than that for the deeper soil moisture in terms of RMSE and G-value. However, the relative performance showed no difference, both with the accuracy ranking as OK<IDW<LR<RK.

**Figure 8:**
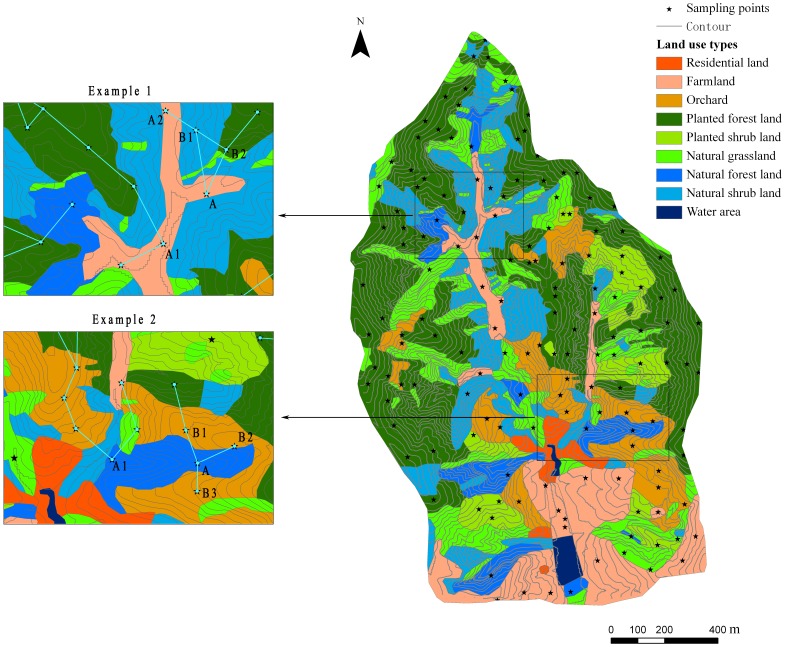
Two examples of the problems when distance-based methods were used in complex terrains.

In addition to the theoretical unsuitability, there are many practical problems if distance-based interpolation methods are used in a complex terrain area. Figure 8 shows two examples of these problems. In the first example, point A (located in the bottom of the valley) was covered with farmland and had high SM, which was similar to nearby points A1 and A2. The points B1 and B2 located on the top and the other side of the hill around the valley, covered with forest/shrub, usually had low SM. However, in the distance-based interpolation methods, the points A1 and A2 would not be chosen to predict the SM in point A because they are not the closest to point A based on the horizontal distance. In contrary, points B1 and B2, which had little similarity with point A, would be determined as the closest points to predict the SM at point A. Thus, a rather unreliable prediction would be produced. Similarly, in the second example, the SM at the bottom of the valley (point A) would be predicted based on the SM on the ridges (points B1, B2 and B3), where the SM condition is usually rather different from that in the valley. On the other hand, the points (point A1)similar to point A would be neglected or only given a very small weight (Fig. 8). Thus, it is difficult to produce a reliable prediction basing on the distance-based interpolation methods in this type of complex terrain, which was confirmed by the performance assessment (Table 3, Table 4).

The LR method showed more effectiveness than the distance-based methods, clearly expressing the impact of the environmental factors on SM, and the prediction was more detailed and accurate than OK and IDW. The variance of SM between different land use types, slopes, and slope aspects is fully displayed in the prediction map (Fig. 4). However, there are some disadvantages to this method. The SM change was fairly sharp at the boundary of the land use types and the junction of the sunny (southern) and shady (northern) slopes. This is inconsistent with the reality, as water is mobile in soil and its spatial change should be gradual at a small scale.

The RK method effectively released the problems of LR, and combined the advantages of LR and kriging model. It not only reserved the impact of the environmental factors on SM obtained from the regression model, but also added the gradually changing property obtained from the kriging model. The prediction map displayed a detailed, reasonable continuous surface, which was more consistent with the reality (Fig. 5). The performance assessment evidently confirmed that this hybrid interpolation method was the most suitable and accurate to the complex terrain in our research.

In conclusion, we suggest that in an area with complex terrain, where the spatial autocorrelation of the interested variable exists only at a small scale and the target variable is significantly correlated with auxiliary variables, the hybrid RK model would perform much better than the distance-based methods in predicting the spatially continuous surface. This conclusion is consistent with the work of Zhu and Lin [23], whose study indicated that RK is more accurate for interpolating soil properties when a strong relationship exists between target soil properties and auxiliary variables as well as when the terrain is more complex.

### Conclusions

The case study of SM spatial interpolation in the hilly gully Loess Plateau shows three main things. First, the distance-based OK and IDW methods performed poorly due to the poor spatial autocorrelation of soil moisture in complex terrain areas, where the environmental impact factors were discontinuous in space at small catchment scale. Second, the LR model performed much better than OK and IDW, and adequately showed the SM difference with the variance of the impact factors. However, the predicted SM changed too sharply near the boundary of the land use types and at the junction of the sunny (southern) and shady (northern) slopes, which was inconsistent with the reality because the soil moisture should change gradually in short distance due to its mobility in soil. Third, the hybrid RK model has evident advantages over the three ordinary approaches for predicting SM in complex terrain area in terms of MAPE, RMSE, and G assessment, with the prediction map being more accurate and realistic.
